# Data on response of *in situ* algal phytoplankton assemblages to micronutrient treatment in small-scale mesocosms for a large hypereutrophic lake

**DOI:** 10.1016/j.dib.2019.103778

**Published:** 2019-02-23

**Authors:** Xiaokai Zhang, Boling Li, Hai Xu, Mona Wells, Boris Tefsen, Boqiang Qin

**Affiliations:** a Department of Biological Sciences, Xi'an Jiaotong-Liverpool University, Suzhou, Jiangsu, 215123, People's Republic of China; b Department of Environmental Science, University of Liverpool, Brownlow Hill, Liverpool, L69 7ZX, United Kingdom; c Taihu Laboratory for Lake Ecosystem Research, State Key Laboratory of Lake Science and Environment, Nanjing Institute of Geography and Limnology, Chinese Academy of Sciences, Nanjing 210008, People's Republic of China; d Freshwater Ecology Group, National Institute of Water and Atmospheric Research, Dunedin 9016, New Zealand

**Keywords:** Copper, Eutrophication, Flow cytometry, Harmful algal bloom, Lake, Micronutrient limitation

## Abstract

This article presents small-scale mesocosm data from “Effect of micronutrients on algae in different regions of Taihu, a large, spatially diverse, hypertrophic lake” [1]. The data is for limitation of the micronutrients boron (B), cobalt (Co), copper (Cu), iron (Fe), and molybdenum (Mo). Data is provided in raw form and includes data from analysis for chlorophyll-a, microscopic counts, and flow cytometry measurement from each sample collected for a total of 255 samples.

**Table:** Specifications table

Subject area	*Biogeochemistry*
More specific subject area	*Freshwater eutrophication*
Type of data	*Figure, tables in xlsx and data in fcs2.0 formats*
How data was acquired	*UV/vis spectroscopy (Cary 60 UV–Vis, Agilent Technologies, California, USA), microscopic counting (ECLIPSE E100, Nikon, Tokyo, Japan), flow cytometry (BD FACSCalibur, Becton Dickinson, California, USA)*
Data format	*Raw*
Experimental factors	*Filtration with glass microfibre filters and ethanol extraction for chlorophyll-a, preservation with Lugol's iodine for microscopic counts*
Experimental features	*Effect of micronutrients on in situ algal phytoplankton in a freshwater lake subject to annual episodes of eutrophication*
Data source location	*Lake Tai, China, 31°28′34.788* ʺ*N, 120°11′39.59ʺE (Meiliang Bay, Station 3), St13, 31°23′11.4″N, 120°17′43.8″E (Gonghu Bay, Station 13)*, *31°12′9.86″N, 120°27′0.6″E (Xukou Bay)*
Data accessibility	*Data are available with this article*
Related research article	*Zhang, X., Li, B., Xu, H., Wells, M., Tefsen, B., Qin, B. 2019. Effect of micronutrients on algae in different regions of Taihu, a large, spatially diverse, hypertrophic lake, Water Research, 21, 500–514.*

**Table:** 

**Value of the data** • The data is on eutrophication, which is a global problem • The data is about micronutrients with anthropogenically altered global biogeochemical cycles • This is a large and exceptionally complete data set covering experiments that are aimed at understanding micronutrient effects on algal growth in a hypereutrophic lake, and the data set may be of use to other investigators in this area who have or are developing specialized approaches to data analysis in this area of eutrophication and anthropogenic impacts

## 1 Data

**Fig. 1:**
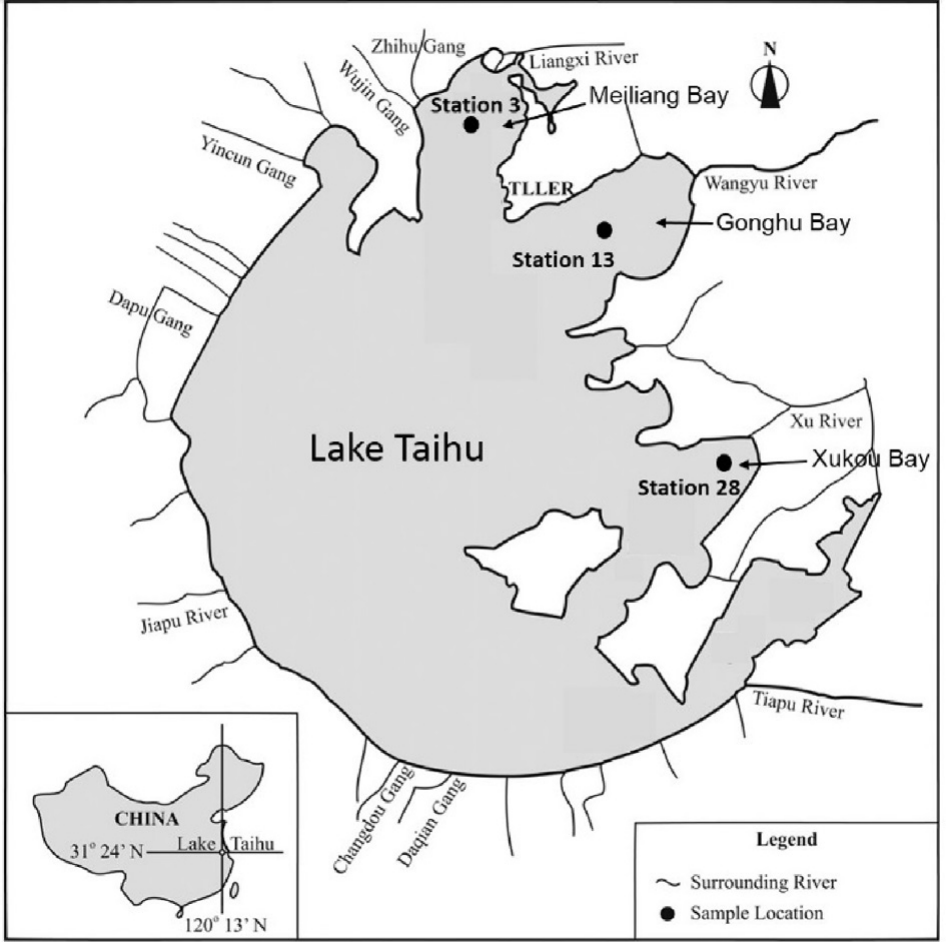
Location of sampling sites in Lake Tai, China.

This data is from a nutrient limitation bioassay (NLB) field mesocosm experiments that are a rapid assessment of nutrient limitation characteristics [2], [3], [4]. The data are for factorial treatments under environmental conditions (e.g. diel cycles, temperature conditions). For the data in this article, water samples were collected from three different parts of Lake Tai, a large hypereutrophic lake in China, including 1) a location actively subject to a harmful algal bloom at the time of sample collection, 2) a location partially impacted by bloom, and 3) a location not affected by an algal bloom (*vide infra* field site data given with Fig. 1 in Section 2.2 below). For this data, water was dosed with the micronutrients B, Co, Cu, Fe, and Mo in the presence and absence of NP (nitrogen and phosphorous together). Controls included no addition (C), N, P and NP. Data is for samples that, after dosing, were deployed in the water at a research station by the lake, and data was collected for three sampling times, t = 0 (initial conditions at the start of mesocosm experiments), t = 2, and t = 4 days. Normally three replicate analyses were performed for each treatment. In all, data is given for chlorophyll-a, microscopic counts, and flow cytometry measurement (FCM) from each sample collected, for a total of 255 samples. The data for chlorophyll-a and microscopic counts are given in two xlsx files. The data for FCM is given in the flow cytometry fcs (2.0) file format. See Supplementary Material.

## 2 Experimental design, materials and methods

### 2.1 Experimental design

The mesocosm experiments followed a factorial design as follows:

- 14 different control, nutrient, and micronutrient treatments (C, N, P, NP, B, B+NP, Co, Co+NP, Cu, Cu+NP, Fe, Fe+NP, Mo, Mo+NP)
- Water with *in situ* phytoplankton collected at three stations
- Mesocosms prepared in triplicate
- Sampling at three time points – for the first time point, data was collected for the original water samples prior to dosing, therefore, t = 0 (initial) samples = 3
- t = 2 and t = 4 samples = 14 × 3 × 3 = 126 at each time point, 84 × 2 for the two time points = 252
- total number of samples represented in data sets = 255

For more context on Lake Tai and issues with eutrophication in Lake Tai, see Qin [5].

### 2.2 Materials

The three sites from which water for mesocosm experiments was collected were in Meiliang Bay (monitoring Station 3), Gonghu Bay (monitoring Station 13), and Xukou Bay (monitoring Station 28), shown in Fig. 1. These locations were, respectively, highly, partially, and not impacted by a harmful algal bloom at the time of sampling in August, 2017. Water was sampled from 0.2 m below the surface into 40-L acid-cleaned polyethylene carboys. For more information about the water quality at the time of sampling for each site see Zhang et al. [1].

### 2.3 Methods

#### 2.3.1 Mesocosm experiments

NLB experiments were carried out immediately after water sample collection and followed the method of Paerl et al. [6] (and supplemental references for interested readers [7], [8], [9]). First, samples were collected for t = 0 analysis. Subsequently, for each NLB treatment, triplicate water subsamples from each respective station were taken from the 40-L samples described in Section 2.2 and placed into 1-L transparent, chemically inert, cubitainers that were trace-metal clean, as described in Xu et al. [7]. Nutrient was then added to cubitainers by spiking with concentrated solution to achieve the final concentrations for each specific component (N – 2.0 mg/L added as KNO_3_; P – 0.20 mg/L added as K_2_HPO_4_·3H_2_O; B – 18 μg/L added as H_3_BO_3_; Co – 1 μg/L added as CoCl_2_·6H_2_O; Cu – 20 μg/L added as CuSO_4_·5H_2_O; Fe – 200 μg/L added as FeSO_4_·7H_2_O; Mo −1 μg/L Mo added as Na_2_MoO_4_·2H_2_O). After nutrient additions, the cubitainers were incubated *in situ* in Taihu near the surface for four days by placing them in a frame at Taihu Laboratory for Lake Ecosystem Research. Each treatment was sampled twice (once at 2 days, once at 4 days) for chlorophyll-a, count, and FCM analysis.

#### 2.3.2 Chlorophyll-a determination and microscopic counting

For the determination of chlorophyll-a, water samples were filtered onto Whatman GF/F glass fiber filters, frozen at −20 °C for no more than 2 days, then the concentration of chlorophyll-a was determined spectrophotometrically after extraction in 90% hot ethanol [10]. Phytoplankton samples were preserved with Lugol's iodine solution for storage and were stored in the dark at room temperature until analysis. Algal objects were counted from observations of samples sedimented in a Sedgwick-Rafter chamber and reported as counts [11]. The phytoplankton species were identified according to Zhou and Chen [12].

#### 2.3.3 Flow cytometry measurement

FCM of single cells was performed using a FACSCalibur (Becton Dickinson, California, USA) with two lasers (argon solid-state, and red diode, excitation at 488 and 635 nm, respectively). For each sample, 800 μL of cell sample was inserted into a 10 mL plastic vial and placed into the flow cytometer with a sample intake speed of 12 μL/min. The sheath fluid was a commercial product (Beckman Coulter Inc., USA), composed of 9.84 g/L Na_2_SO_4_, 4.07 g/L NaCl and 0.11 g/L procaine hydrochloride, pH 7.0, delivered through a 150 μm nozzle at 4.5 psi. Measurements included forward scatter (FSC), side scatter (SSC) and four fluorescence channels: green fluorescence (FL1, 530/30 nm bandpass), yellow fluorescence (channel FL2: 585/42 nm bandpass), red fluorescence (channel FL3: 670 nm/longpass) and orange fluorescence (FL4: 661/16 nm bandpass). For FSC and SSC the amplification gain was set at 1 and measured in the linear mode. For fluorescence channels the amplification gain was set at 1 and measured in the log-mode. Acquisition was set to capture 50,000 total events for each sample.
